# Small but protective social capital against suicide ideation in poor communities: A community-based cross-sectional study

**DOI:** 10.1097/MD.0000000000022905

**Published:** 2020-10-30

**Authors:** Minjae Choi, Myung Ki, Paul S.F. Yip, Jungyoun Park, Areum Song, Weon Young Lee, Jong-Woo Paik, Jiseun Lim

**Affiliations:** a Department of Public Health, Korea University; b Department of Preventive Medicine, Korea University College of Medicine, Seoul, Republic of Korea; c Hong Kong Jockey Club Centre for Suicide Research and Prevention, The University of Hong Kong, Pokfulam, Hong Kong SAR, China; d Department of Preventive Medicine, Chung-Ang University College of Medicine, Dongjak-Gu; e Department of Psychiatry, College of Medicine, Kyung Hee University, Dongdaemun-gu, Seoul; f Department of Preventive Medicine, Eulji University, Daejeon Jung-gu, Daejeon, Republic of Korea.

**Keywords:** participation, resilience, social capital, socioeconomic status, suicidal ideation, trust

## Abstract

Coupled with the lowest level of social connectedness, South Korea has the highest suicide rate among the Organization for Economic Co-operation and Development countries. A possible link between community and suicide is social capital imprinted in social connectedness. This study explores whether social capital is protective against suicide ideation in relation to the poverty level of communities, and whether the associations are specific to certain elements of social capital.

A total of 908 participants were included to assess cross-sectional association of social capital at individual level with suicide ideation by comparing between poor (government-leased apartments) and non-poor communities (nongovernment-leased apartments). Logistic regression analyses were performed to examine various social capital dimensions in relation to suicide ideation.

Suicide ideation was far higher among those living in the poor communities (poor communities 12%; non poor communities 6.3%) and the level of social capital was lower in the poor communities. Nevertheless, the protective effect of social capital, in particular, the cognitive dimension against suicide ideation was demonstrated only in the poor communities (eg, odds ratio = 0.27, 95% confidence interval: 0.12–0.58 for trust in the poor communities). Low income was significantly associated with suicide ideation only in the poor communities, but depression and resilience were associated with suicide ideation both in the poor and non-poor communities.

To increase the reliability of the results, established measures based on relevant literature were utilized, but measures on bridging social capital and social network might have relatively low reliability.

As to protection against suicide ideation, the extent of reliance on social capital was higher in poor communities than in non-poor communities, in particular, the cognitive dimension was likely to activate in this regard.

## Abstract 2

Supplemental Digital Content is available in the text

## 1 Introduction

South Korea has the highest suicide rate among the Organization for Economic Co-operation and Development countries.^[1]^ In particular, in 2018, the elderly population (≥65) leads the tragic epidemic with a prevalence of 48.6 per 100 thousand compared to 26.9 among other age groups (15–64 years). In conjunction with the growth of the elderly population in Korea, the far higher suicide rate among the elderly population poses an increasingly important social issue. Given the fact that Korea records the lowest level of social support among Organization for Economic Co-operation and Development countries in recent years^[2]^ and that social support has protective effects on suicide (ideation),^[3]^ the epidemic of suicide in Korea seems to be linked to its loose social connectedness.

Social capital, referring to the acquisition of resources embedded in social connectedness,^[4]^ has been recognized to influence suicide^[5,6]^ as well as a variety of other health outcomes such as mortality and mental health.^[7,8]^ In the absence of other forms of capital^[9–11]^ such as cultural and economic capital, social capital is recognized to have more pronounced protective effects on the health of the poor than the non-poor. However, some studies where social capital was mainly specified at the individual level showed that the protective effects of social capital on general health^[12]^ and suicide prevention^[6]^ may not be equal among subgroups, or even increase the risk of spreading undesirable consequences^[13]^ as adverse social interaction may bring negative impact on health.^[14]^ A recent review also reported that social capital functions either as a buffer or a dependency relationship against adverse socioeconomic circumstances in influencing health, although more evidence supported the former.^[15]^ Thus, social capital has both moderating and destructive potential under adverse socioeconomic circumstances, but uncertainty would depend on which elements of social capital are more significant in socioeconomically disadvantaged communities and how they are linked.

Social capital or social relations in the broad sense, is a multi-dimensional concept (eg, social support, network, trust, reciprocity, and participation), and the use of various measures contributes to the inconsistent demonstration of the effects of social capital. Some previous studies using a composite score such as the state social capital index,^[8]^ the summative social capital score,^[16]^ the social fragmented index,^[17]^ or the social embeddedness scale^[18]^ monitored the overall status of social capital, but this masked substantial variations across different items. Other studies focusing on selected dimensions of social capital such as trust^[19]^ or social support^[3]^ were limited to represent comprehensive dimensions of social capital.^[5]^ Similarly, most Korean studies on the association between social capital and suicide focused on 1 or 2 dimensions (eg, social support, social participation, and trust, and reported positive association with suicide (ideation).^[20,21]^ However, studies including comprehensive dimensions of social capital showed that its association with mental health and suicide varies according to the types of social capital.^[22]^ In a cross-sectional ecological study,^[23]^ social trust, but not social participation or helpfulness, was linked with suicide rate. A study on an Australian rural population^[24]^ nonetheless showed that social support but not social network or a sense of community was associated with suicide ideation. A few Korean studies^[25,26]^ simultaneously included various dimensions of social capital, where, for example, trust, but not social participation, was associated with suicide ideation among the elderly group.^[25]^ Thus, conceptual differentiation of social capital into several dimensions calls for assessing the association between social capital and suicide, but evidence remains unclear as to whether some aspects of social capital are more influential than others.

Further, social capital was differentiated into 2 aspects; bonding and bridging social capital.^[4,27]^ Bonding social capital is defined as horizontal connections among members of similar networks and bridging social capital as social connection between individuals in dissimilar groups. Some studies clarified that what ensures the beneficial effects of social capital for the disadvantaged population is bridging, but not bonding social capital.^[6,28]^ Thus, inclusion of both types of social capital would be necessary to better understand the potential mechanism of the interactions between social capital and adverse socioeconomic circumstances. In Korea, large apartment blocks (the typical form of residence in urban area) are developed separately for the poor and non-poor people. Large apartment blocks for the poor are mostly public-leased and are clearly demarcated from the outside, facing problems of stigmatization and social exclusion. These blocks are considerably self-contained and homogeneous, representing an independent community, isolated and dissimilar to the surrounding general residential area in terms of social identity and power relations. In the current study, both public-leased housing blocks and general apartment blocks were separately included to provide a unique opportunity for a natural experiment in the comparison of social capital between the poor and non-poor communities.

Based on the samples from the contrasting poor and non-poor communities, the current study aims to examine

(1) whether the association of social capital with suicide ideation is protective, in particular, in relation to the poverty level of communities; and
(2) whether the association is related to a specific dimension of social capital with simultaneous inclusion of various constructs of social capital (ie, social participation, trust, reciprocity, network, and bridging social capital).

## 2 Methods

### 2.1 Study population

A 2-stage convenience sampling was used to recruit the study participants: communities as a primary sampling unit and individuals as a secondary unit. Based on the information of public-leased apartments provided by the Seoul Housing & Communities Corporation, 2 largest public-leased apartment blocks in each district were selected from the 24 blocks in Dongdaemoon and 6 blocks in the Jungrang-gu district in Seoul. Non-poor communities were selected from the neighboring general apartment blocks in Dongdaemoon-gu, to enhance comparability of economic status between poor and non-poor apartment blocks, while minimizing differences in other regional characteristics. Samples of 607 (47%) participants were selected among the 1294 elderly population (≥60 years) in the poor communities, and another sample of 301 (7%) participants were selected among 4400 elderly population in the non-poor communities. This satisfied a required sample size,^[29]^ calculated based on difference in prevalence of elderly suicide ideation between poor community (12.14%) and non-poor (4.06%) reported in a prior Korean study.^[30]^ Then, any eligible individuals were contacted mostly at home with prior announcements and flyers providing them with information on the survey. After excluding an individual with missing data (n = 1), the final sample of 908 was included in the analyses. Ethical approval for data collection was obtained from the Institutional Research Board at Korea University (approval number: 1040548-KU-IRB-17–193-A-2).

### 2.2 Measure

For suicide ideation, the participants were asked to respond (yes or no) to a question on suicidal thoughts: “Have you ever felt like dying or killing yourself during the previous 12 months?.”

Based on the appraisal of previous publications, relevant dimensions, and reliable measures of social capital were identified and generated (Supplementary Table 1). Social network was assessed by totaling the responses to 3 questions on the quantity of contact per month with family and relatives, neighbors or friends.

Then, it was categorized into 2 groups (low and high) based on the median value of the summary score. Social trust was measured using the responses (very good, good, fair, bad, and very bad) to 2 questions (“In general, do you think that your neighbors can be trusted?” and “Do you think your community has a culture that neighbors help you with family events?”) and according to the summary score, the respondents were subdivided into 2 groups. Reciprocity was assessed using the responses on the Likert scale to the questions: “Among the people in each of the following 3 categories (family and relatives, neighbors and friends), how many of them will definitely help you upon your request?.”^[31]^ The high and low reciprocity categories were defined based on a median value of summary score. Social participation was measured by asking whether the respondents regularly participate in five types of activities (religion, friendly meeting, senior citizen center, leisure, and voluntary organization). Those who responded positively to 1 or more of the 5 activities were classified as the high social participation group and others as the low social participation group. In this study, a single question on the general relationship outside a community was used to broadly imply bridging social capital: “Do you work or interact with other groups outside your apartment block?” This question was based on the Integrated Questionnaire for the Measurement of Social Capital developed by the World Bank (“Does this group work or interact with other groups with different/similar goals outside the village/neighborhood?”)^[32]^ with revision on the wording to consider the focus of the study unit (“you” instead of “group”) and the context (“an apartment block” instead of “village/neighborhood”). The 3 responses (“no,” “sometimes,” or “yes”) were dichotomized into no versus sometimes and yes. Resilience was measured using the responses to the questions on adaptation to change and on recovery after hardship; high and low resilience were classified based on the summary score.

The sociodemographic covariates considered in the analyses were age, gender, equivalised household income, and marital status. Equivalized household income was calculated by dividing the total household income by the square root of the number of household members based on the Luxembourg Income Study.^[33]^ Then, median equivalized household income (1,125,000 Korean Won) was obtained from the distribution of the entire study population. To examine mental health status, the Geriatric Depression Scale was used where a score of 8+ is the cut-off for depression.^[34]^ To assess their general health status, the respondents were asked to self-rate their health. All of these variables were dichotomized.

### 2.3 Statistical analyses

The prevalence of each of the components of social capital, demographic, socioeconomic, and health-related variables were calculated. To compare the associations between social capital and suicide ideation between the poor and non-poor communities, a bivariate distribution was examined, and covariate-adjusted odds ratios (ORs) using logistic regression models computed. All analyses were conducted separately for 2 contrasting communities.

Three models were used to assess the influence of covariates on the magnitude of the associations. Model 1 was unadjusted to account for basic differences across the 3 communities. In Model 2, income, along with age and gender, was adjusted to assess the influence of the socioeconomic factors. In Model 3, marital status and health-related factors (subjective health and depression) were adjusted in addition to those in Model 2. Social capital variables were entered in the 3 models separately to avoid multi-collinearity problem due to simultaneous modeling of highly inter-correlated variables.^[35]^ The age distributions of the 2 communities differed and, therefore, sample weights were developed for indirect age-standardization using the total study sample. To confirm the interaction effects between the social capital variables and socioeconomic circumstances on suicide ideation, the Synergy Index (“SI”) was calculated.^[36]^ All statistical analyses were conducted using SAS version 9.4.

## 3 Results

The general characteristics of individuals differed between the poor communities and the non-poor communities. The prevalence of suicide ideation was higher in the poor than the non-poor communities (12.0% vs 6.3%). The prevalence of social network and reciprocity in the non-poor communities were almost 2-fold higher than in the poor communities. In contrast, the prevalence of bridging social capital was higher in the poor communities. The other social capital variables showed no obvious pattern across the communities (Table 1).

**Table 1:**
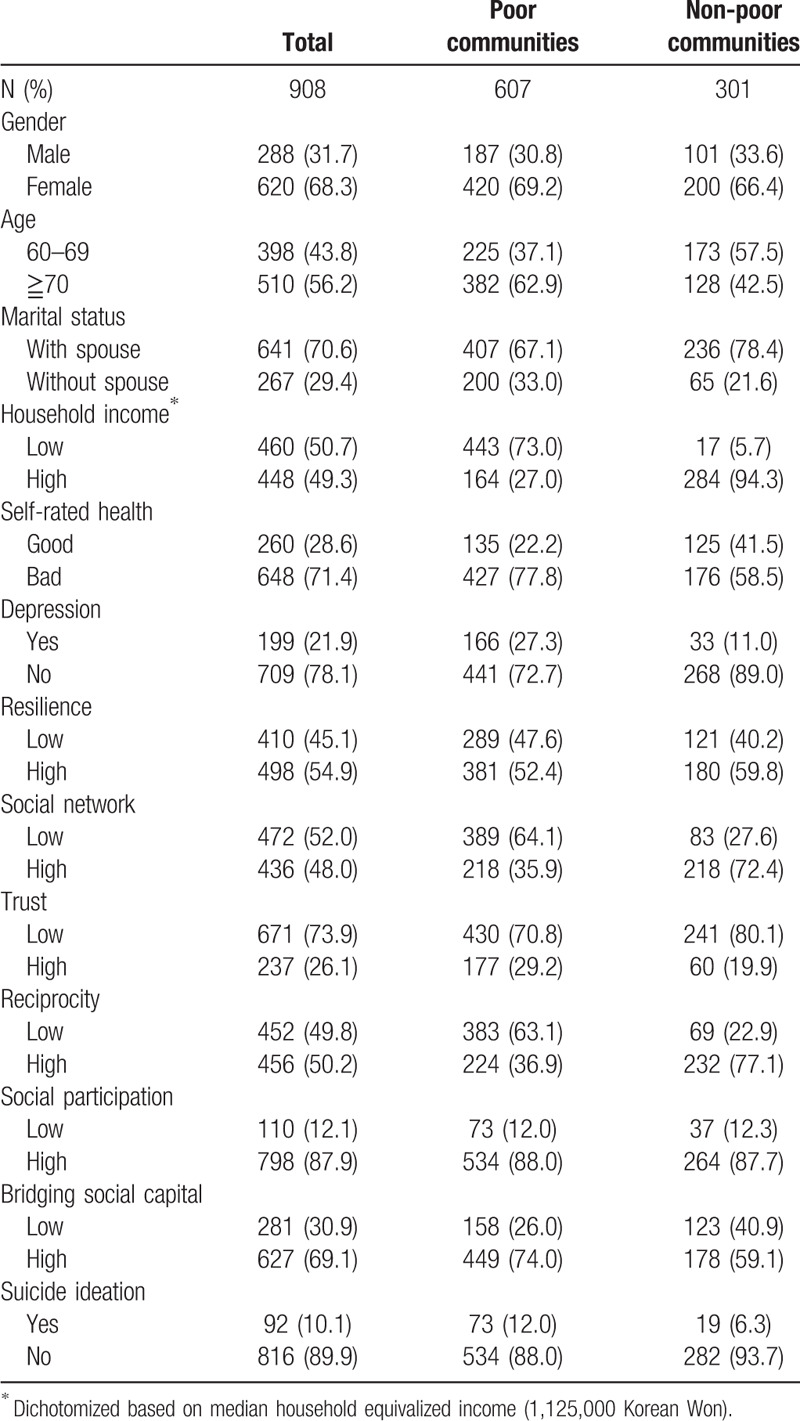
General characteristics of participants in the poor and non-poor communities.

The bivariate associations between the sociodemographic, health-related, and social-capital variables and suicide ideation in the poor and non-poor communities are shown in Table 2. In general, adverse sociodemographic and health-related factors (ie, without a spouse, poor self-rated health, and depression) were associated with suicide ideation. Similarly, except for social network and bridging social capital, a low level of social capital across measures was associated with a high level of suicide ideation, but this was mostly observed in the poor communities. The difference in the prevalence of suicide ideation between the high- and low-social capital groups was generally larger in the poor communities than in the non-poor communities (ie, 5.1% [high trust] and 14.9% [low trust] in the poor communities vs 3.3% and 7.1%, respectively, in the non-poor communities).

**Table 2:**
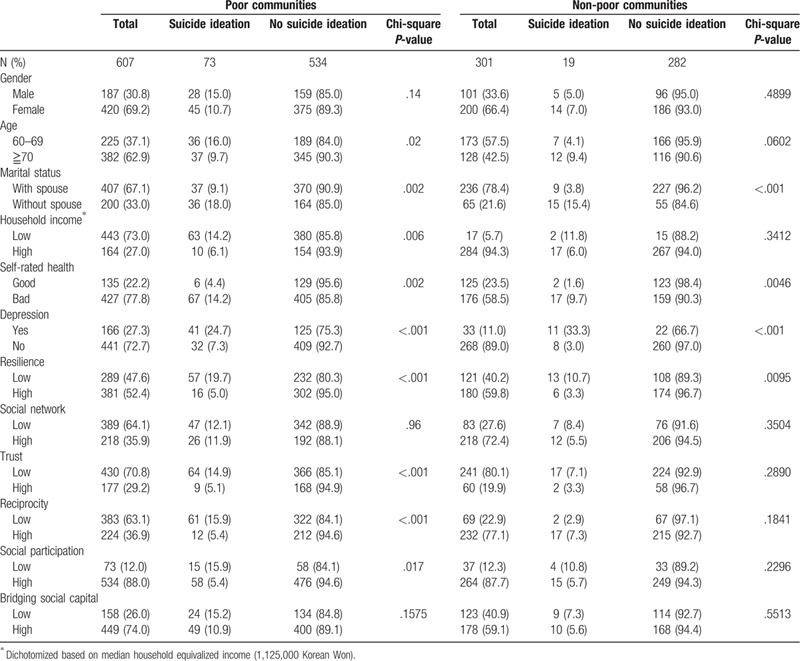
Bivariate association between sociodemographic and social capital variables and suicide in the poor and non-poor communities.

**Figure 1:**
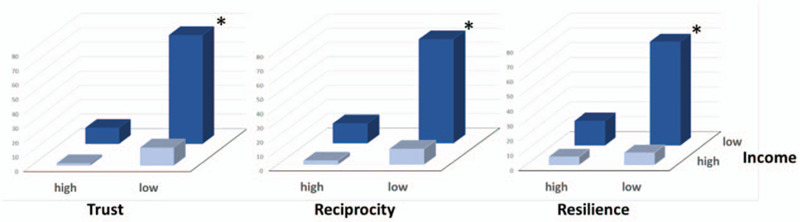
SES-social capital interaction association on suicide ideation in the poor communities. ^∗^Signigncant interaction effect (*P*-value < .05) between social captial and SES on suicide ideation. SES = socioeconomic status.

Multivariable associations between the measures of social capital and suicide ideation are shown in Table 3. The associations were only presented in poor communities; trust and reciprocity were significantly associated with suicide ideation throughout all the 3 models (odds ratio = 0.27; 95% confidence interval [CI]: 0.12–0.58 in trust and OR = 0.24; 95% CI: 0.12–0.47 in reciprocity); associations of social participation and bridging social capital were present but attenuated in some models. Social network; however, was not associated with suicide ideation. Association of resilience with suicide ideation remained significant, but the association in the non-poor communities disappeared in Model 3. Low income was associated with suicide ideation in the poor communities, though again, it disappeared in Model 3. Depression was associated with suicide ideation in both the poor and non-poor communities but the magnitude of association was far larger in the non-poor communities. Interaction effects between social capital and socioeconomic status on suicide ideation were also identified (Fig. 1) in the poor communities (trust: SI = 0.67, 95% CI: 0.47–0.96, reciprocity: SI = 0.58, 95% CI: 0.41–0.79, resilience: SI = 0.66, 95% CI: 0.45–0.97 and social participation: SI = 0.51, 95% CI: 0.40–0.65) (Supplementary Table 2).

**Table 3:**
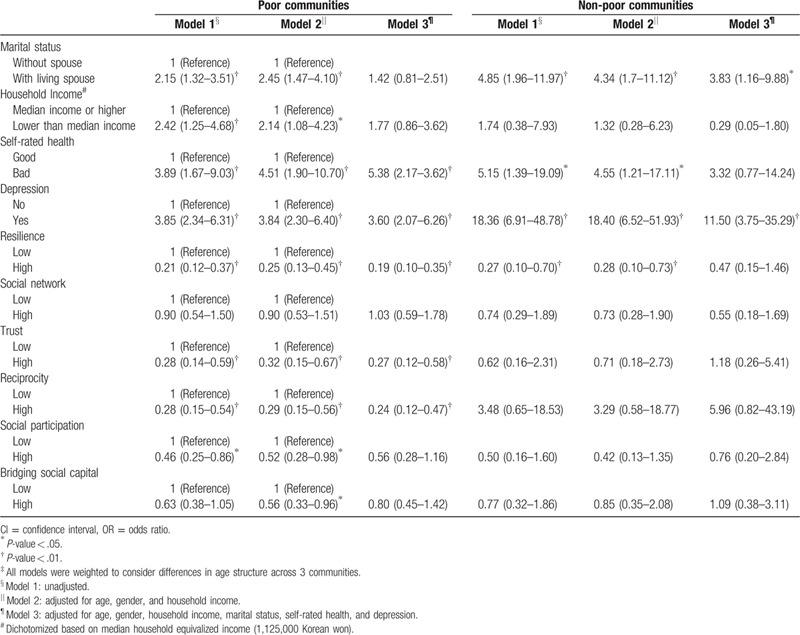
Multivariate association (OR [95% CI])^‡^ between sociodemographic and social capital variables and suicide ideation in the poor and non-poor communities.

## 4 Discussion

The prevalence of suicide ideation was higher in the poor than in the non-poor communities. In general, the poor communities had lower levels of social capital but higher levels of trust and bridging social capital. Specific dimensions of social capital (eg, trust and reciprocity) were independently associated with suicide ideation but only reflected in the poor communities. Social network was not associated with suicide ideation. Resilience was consistently protective against suicide ideation in both the poor and non-poor communities. Depression appeared a stronger association with suicide ideation in the non-poor communities, while low income was associated to suicide ideation in the poor communities, though attenuated.

### 4.1 Methodological consideration

This study has several strengths. First, social capital was differentiated into several dimensions and this enabled examination of separate concepts of social capital on suicide ideation and simultaneous assessment of conceptual pairs (eg, bonding vs bridging and cognitive vs structural elements). Second, inclusion of the contrasting communities allowed exploration of the association between social capital and suicide ideation in relation to individual- and community-level poverty. This also facilitated a detailed examination of how social capital operated differently in the poor and non-poor communities. Nevertheless, due to the same reason that the present sample was selected from a few communities, the available data were limited to a small number of individuals in some subgroups (eg, poor people living in a non-poor communities).

This study also has several limitations. First, social capital is a broad concept, the results may rely on the number of social capital measures defined in a study.^[37]^ To increase the reliability of the results in the current study, established measures based on relevant literature were utilized, but some measures might have relatively low reliability. In particular, the bridging dimension of social capital was measured using a response to a single question in the relationship outside the apartment block. This may correspond to a central concept of bridging social capital, but the measure was narrowly defined to represent various bridging types on the relationship across divergent segments of society such as socioeconomic levels, religion, political orientation, age, and occupation.^[38]^ Likewise, social network was defined as the frequency of contact with friends, neighbors, and relatives but the quality of network was not inquired. Also, colleague networks, which is often regarded as a main form of network in Korea, were not included. Second, this study was based on a cross-sectional design, this limits the causal inference that refers to the direction between social capital and suicide ideation. Poor health reportedly increased the risk of low social capital,^[4]^ implying that the relationship between social capital and suicide ideation was bi-directional, and part of the association between social capital and suicidal ideation might be attributed to the reversed causal direction; that is, suicide ideation preceded and endorsed low levels of social capital. Therefore, a longitudinal approach is warranted to consider the situation when suicide ideation precedes low social capital. Third, social capital was specified as an individual attribute, though the features of social capital lie in the area-level as well.^[39]^ Despite the limited capacity of this type of approach, individual-level studies as the present, were able to highlight the associations between social capital, poverty, and suicide operating as individual characteristics.^[40]^ Fourth, another limitation concerns the unmeasured confounding factors such as anxiety and alcohol abuse. Alcohol drinking even at a moderate drinking level was reported to be a strong risk factor for suicide in Korea,^[41]^ therefore, a future study is required to assess the association between social capital and alcohol consumption levels in predicting suicides. Lastly, as there was restriction to obtaining the list of residents, the sampling was not conducted in a probabilistic or random manner. This approach may be subjected to a potential problem in representing a population. However, as the available samples from the poor and non-poor communities were relatively large (7%–50% of the total population) with low decline rates (3%–8%) to the survey request, the data can be considered to be generalizable.

### 4.2 Comparison with previous studies

In the current study, the level of social capital appeared to differ by dimensions between the poor and non-poor communities. However, those living in the poor communities are more often related to formal networks, and actual social capital is generally more restrictive in the poor communities. For example, the frequency of social participation was similar in the poor and non-poor communities, and the residents of the poor communities participated more frequently in activities in senior citizen centers but less frequently in leisure and voluntary activities (Supplementary Table 3). Likewise, bridging social capital, which was higher among the residents of the poor communities may reflect aid-based relationships, as the senior citizen centers and welfare centers frequently provide basic services such as meals to the poor elderly persons. As the residents of the poor communities are more likely to rely on survival-oriented assistance, this may appear to increase their social capital.^[42]^ However, in contrast to previous findings, in the current study, social trust was higher in the poor communities.^[43]^ Given that the present 2 questions of trust are primarily related to credit on the neighborhoods, the findings may reflect the possibility that the affluent people are less concerned about their relationships with neighbors, which may reduce the level of trust among the residents of the non-poor communities.^[44]^

The association of social capital with suicide ideation was demonstrated only in the poor communities, suggesting that the residents of those communities are more dependent on social capital. Previous studies on the impact of social capital on health support this notion that the impact of social capital was greater or only detected in deprived areas,^[45]^ ethnic minorities,^[12]^ and lower socioeconomic status (“SES”) groups.^[6,43]^ This may be because social capital compensates for insufficient material resources in poor communities^[15]^ and/or social relationships in poor communities are more cohesive and more likely to provide effective support necessary to maintain mental health.^[43]^

An interaction effect between social capital and SES on suicide ideation was detected in the poor communities where the association between social capital and suicide ideation was strengthened among those with a low income.^[13,46]^ Also, some evidence was included by differentiating community level poverty, that the moderating role of social capital on the association between SES and suicide ideation was marked in the poor communities but minimal in the non-poor communities. This finding further supports that when other forms of capital are less available, social capital may be more important among those in poverty both at the individual- and community-levels.

Some dimensions of social capital, however, did not show protective associations with suicide ideation, while trust and reciprocity exerted a marked protective effect. This finding sheds some light on the distinction between the cognitive and structural dimensions of social capital. Cognitive social capital is typically defined as subjective perceptions of social relations such as trust, reciprocity, and norm, while structural social capital reflects directly observable measures such as social network and social participation.^[47,48]^ Similar to previous studies,^[22,48]^ and including a review,^[49]^ it is discovered that cognitive social capital is more beneficial in preventing suicide than structural social capital. Thus, the influence of social capital on suicide ideation in the poor communities may be more related to perceived rather than actual levels of social connectedness.

Resilience showed protective effects against suicide ideation both in the poor and non-poor communities. This is consistent with previous studies where regardless of the characteristics of the study population (eg, adolescents, the elderly, trauma patients, depressed persons, and rural residents),^[50–52]^ the associations of resilience on suicidality (eg, suicide ideation and suicide attempts) in Korea^[50,51]^ and elsewhere^[53,54]^ were observed. In these studies, the magnitude of the association of resilience has been particularly underscored, suggesting that psychological factors played important roles in preventing suicides in Korea. This is also consistent with the present finding that protection against suicide ideation is mediated primarily by the cognitive rather than the structural dimensions of social capital.

In the current study, low income was associated with suicide ideation, probably because low SES can lead to psychological distress and ultimately suicide ideation.^[55]^ Further, similar to previous studies where the association between low income and suicide is stronger in deprived than in non-deprived areas,^[56]^ low income was significantly associated with suicide ideation only in the poor communities, though it attenuated. Interestingly, depression was associated with suicide ideation in both communities. This is consistent with the well-known findings that suicidality could be attributable to being depressed, and in the current study, only a smaller magnitude of this association was shown in the poor communities.^[57]^ This may be because mental health issues alone are typically insufficient to induce suicides. Other stressors, for example, debt and physical illnesses, are usually also present^[58]^; these factors are more frequently encountered in the poor communities.^[57]^ This suggests that the relative importance between SES versus psychological factors of suicide risk differs between the poor and non-poor communities.

## 5 Conclusions

In the poor communities, social capital may be low in general, while it can be suicide protective among those with strong ties. This finding should be viewed as emphasizing the importance of both social capital and economic progress. The associations of social capital with suicide ideation were linked to specific dimensions and cognitive social capital such as trust and reciprocity, and likely to function in the poor communities. Further, social capital exerted a moderating role on the association between income and suicide ideation, in particular, in the poor communities.

## Author contributions

**Conceptualization:** Minjae Choi, Myung Ki.

**Formal analysis:** Minjae Choi.

**Methodology:** Myung Ki.

**Writing – original draft:** Minjae Choi, Myung Ki.

**Writing – review & editing:** Paul Siu Fai Yip, Jungyoun Park, Areum Song, Weon Young Lee, Jong-Woo Paik, Jiseun Lim.

## Supplementary Material

Supplemental Digital Content

## Supplementary Material

Supplemental Digital Content

## Supplementary Material

Supplemental Digital Content
