# Supplementary material for: Small but protective social capital against suicide ideation in poor communities: A community-based cross-sectional study
Source: Medicine (Baltimore). 2020 Oct 30;99(44):e22905. doi: 10.1097/MD.0000000000022905 (PMC7598880; doi:10.1097/MD.0000000000022905)
Supplement: Supplemental Digital Content [file medi-99-e22905-s001.docx]

Supplementary table 1. The dimensions and measures of social capital constructs in this study with reference

| Dimensions | Measures | Reference |
| --- | --- | --- |
| Trust | -Do you think that your neighbors can be trusted in general? (very good, good, fair, bad, very bad)  -Do you think that your community has a culture that neighbors help you with family events? (very good, good, fair, bad, very bad) | Integrated Questionnaire for the Measurement of Social Capital (SC-IQ), World Bank (2004) ^[1]^ |
| Reciprocity | -Among people in each of the following five categories (family, relatives, neighbors, friends), how many will definitely help you upon your request? (none, a few, some, most, all) | Personal Social Capital Scale, Chen et al. (2008) ^[2]^ |
| Social network | -With how many of people in each of the following categories (family/relatives, neighbors, friends) do you keep a routine contact? (None, less than one time per month, one time per month, two-three times per month, one time per a week, two-three times per a week, more than four times per a week) | Personal Social Capital Scale, Chen et al. (2008) ^[2]^ |
| Social participation | -In the last 12 months have you been an active member of any of the following types of groups in your community? (community association, religious group, credit group, sports/leisure group, voluntary organization, other: specify) | Shortened and adapted Social Capital Assessment Tool (SASCAT), De Silva et al. (2006) ^[3]^ |
| Bridging social capital | -Do you work or interact with other groups outside your apartment? (no, sometimes, yes) | Integrated Questionnaire for the Measurement of Social Capital (SC-IQ), World Bank (2004) ^[1]^ |

**Reference**

[1] Grootaert C, Narayan D, Jones VN, Woolcock M. Measuring social capital: An integrated questionnaire: The World Bank, Washington; 2004.

[2] Chen X, Stanton B, Gong J, Fang X, Li X. Personal Social Capital Scale: an instrument for health and behavioral research. Heal Educ Res 2008;24:306-17.

[3] De Silva MJ, Harpham T, Tuan T, Bartolini R, Penny ME, Huttly SR. Psychometric and cognitive validation of a social capital measurement tool in Peru and Vietnam. Soc Sci Med 2006;62:941-53.
