# Supplementary material for: Small but protective social capital against suicide ideation in poor communities: A community-based cross-sectional study
Source: Medicine (Baltimore). 2020 Oct 30;99(44):e22905. doi: 10.1097/MD.0000000000022905 (PMC7598880; doi:10.1097/MD.0000000000022905)
Supplement: Supplemental Digital Content [file medi-99-e22905-s002.docx]

Supplementary table 2 Interaction effect between elements of social capital and socio-economic status in thepoor and non-poor communites using Synergy index

|  | Poor community  Interaction effect (95% CI) | Non-poor community  Interaction effect (95% CI) |
| --- | --- | --- |
| Social network | 1.56(0.24-9.82) | 0.40(0.09-1.79) |
| Trust | 0.67(0.47-0.96)^*^ | 0.23(0.01-3.80) |
| Reciprocity | 0.57(0.41-0.79)^*^ | 2.39(0.13-44.88) |
| Resilience | 0.66(0.45-0.97)^*^ | 0.18(0.03-1.26) |
| Social participation | 0.51(0.40-0.65)^*^ | 0.51(0.15-1.74) |
| Bridging network | 0.71(0.34-1.48) | 0.59(0.13-2.69) |

^*^ p-value <0.05
