# Supplementary material for: Small but protective social capital against suicide ideation in poor communities: A community-based cross-sectional study
Source: Medicine (Baltimore). 2020 Oct 30;99(44):e22905. doi: 10.1097/MD.0000000000022905 (PMC7598880; doi:10.1097/MD.0000000000022905)
Supplement: Supplemental Digital Content [file medi-99-e22905-s003.docx]

Supplementary table 3 Detailed general characteristics of participants in the poor and non-communities

|  | Total | Poor communities | Non poor community |
| --- | --- | --- | --- |
| N (%) | 908 | 607 | 301 |
| Gender |  |  |  |
| Male | 288(31.7) | 187(30.8) | 101(33.6) |
| Female | 620(68.3) | 420(69.2) | 200(66.4) |
| Age |  |  |  |
| ≧ 69 | 398(43.8) | 225(37.1) | 173(57.4) |
| 70-79 | 407(44.8) | 315(51.9) | 92(30.6) |
| 80 ≧ | 103(11.4) | 67(11.0) | 36(12.0) |
| Marital status |  |  |  |
| With spouse | 641(70.6) | 405(66.7) | 236(78.5) |
| Cohabitating | 2(0.2) | 2(0.3) | 0(0.0) |
| Divorced | 32(3.5) | 16(2.6) | 16(5.3) |
| Widowed | 224(24.7) | 180(29.7) | 44(14.6) |
| Separated | 7(0.8) | 3(0.5) | 4(1.3) |
| Not married | 2(0.2) | 1(0.2) | 1(0.3) |
| Household income |  |  |  |
| ≦ median income | 460(50.7) | 443(73.0) | 17(5.7) |
| > median income | 448(49.3) | 164(27.0) | 284(94.3) |
| Self-rated health |  |  |  |
| Yes | 260(28.6) | 135(22.2) | 125(23.5) |
| No | 648(71.4) | 472(77.8) | 176(58.5) |
| Depression |  |  |  |
| Yes | 199(21.9) | 166(27.3) | 33(11.0) |
| No | 709(78.1) | 441(72.7) | 268(89.0) |
| Social network |  |  |  |
| Family & relatives | 481(52.9) | 257(42.3) | 224(74.4) |
| Neighbor | 436(48.0) | 231(38.1) | 205(68.1) |
| Friends | 319(35.1) | 120(19.8) | 199(66.1) |
| Trust |  |  |  |
| Neighbor trust | 450(49.6) | 315(51.9) | 135(44.9) |
| Family event support | 277(30.5) | 208(34.3) | 69(22.9) |
| House environment | 405(44.6) | 215(35.4) | 190(63.1) |
| Reciprocity |  |  |  |
| Family(relatives) | 287(31.6) | 96(15.8) | 191(63.5) |
| Neighbor | 167(18.4) | 75(12.4) | 92(30.6) |
| Friend | 224(24.7) | 88(14.5) | 136(45.2) |
| Resilience |  |  |  |
| Adaptation of change | 457(50.3) | 293(48.3) | 164(54.5) |
| Recover of hardship | 356(39.2) | 211(34.8) | 145(48.2) |
| Social participation |  |  |  |
| Religion | 298(32.8) | 208(34.3) | 90(29.8) |
| Social activity | 601(66.2) | 370(61.0) | 231(76.6) |
| Senior community center | 425(46.8) | 355(58.5) | 70(23.3) |
| Leisure | 277(30.5) | 98(16.1) | 179(59.6) |
| Charity | 39(4.3) | 18(3.0) | 21(7.0) |
| Bridging network |  |  |  |
| Yes | 627(69.1) | 449(74.0) | 178(59.1) |
| No | 281(30.9) | 158(26.0) | 123(40.9) |
| Suicide ideation |  |  |  |
| Yes | 92(10.1) | 73(12.0) | 19(6.3) |
| No | 816(89.9) | 534(88.0) | 282(93.7) |
